# Population structure and genomic inbreeding in nine Swiss dairy cattle populations

**DOI:** 10.1186/s12711-017-0358-6

**Published:** 2017-11-07

**Authors:** Heidi Signer-Hasler, Alexander Burren, Markus Neuditschko, Mirjam Frischknecht, Dorian Garrick, Christian Stricker, Birgit Gredler, Beat Bapst, Christine Flury

**Affiliations:** 1 School of Agricultural, Forest and Food Sciences, Bern University of Applied Sciences, Zollikofen, Switzerland; 2 Agroscope, Swiss National Stud Farm, Avenches, Switzerland; 3 Qualitas AG, Zug, Switzerland; 4 Massey University, Hamilton, New Zealand; 5 agn Genetics, Davos, Switzerland

## Abstract

**Background:**

Domestication, breed formation and intensive selection have resulted in divergent cattle breeds that likely exhibit their own genomic signatures. In this study, we used genotypes from 27,612 autosomal single nucleotide polymorphisms to characterize population structure based on 9214 sires representing nine Swiss dairy cattle populations: Brown Swiss (BS), Braunvieh (BV), Original Braunvieh (OB), Holstein (HO), Red Holstein (RH), Swiss Fleckvieh (SF), Simmental (SI), Eringer (ER) and Evolèner (EV). Genomic inbreeding (*F*_ROH_) and signatures of selection were determined by calculating runs of homozygosity (ROH). The results build the basis for a better understanding of the genetic development of Swiss dairy cattle populations and highlight differences between the original populations (i.e. OB, SI, ER and EV) and those that have become more popular in Switzerland as currently reflected by their larger populations (i.e. BS, BV, HO, RH and SF).

**Results:**

The levels of genetic diversity were highest and lowest in the SF and BS breeds, respectively. Based on *F*_ST_ values, we conclude that, among all pairwise comparisons, BS and HO (0.156) differ more than the other pairs of populations. The original Swiss cattle populations OB, SI, ER, and EV are clearly genetically separated from the Swiss cattle populations that are now more common and represented by larger numbers of cows. Mean levels of *F*_ROH_ ranged from 0.027 (ER) to 0.091 (BS). Three of the original Swiss cattle populations, ER (*F*_ROH_: 0.027), OB (*F*_ROH_: 0.029), and SI (*F*_ROH_: 0.039), showed low levels of genomic inbreeding, whereas it was much higher in EV (*F*_ROH_: 0.074). Private signatures of selection for the original Swiss cattle populations are reported for BTA4, 5, 11 and 26.

**Conclusions:**

The low levels of genomic inbreeding observed in the original Swiss cattle populations ER, OB and SI compared to the other breeds are explained by a lesser use of artificial insemination and greater use of natural service. Natural service results in more sires having progeny at each generation and thus this breeding practice is likely the major reason for the remarkable levels of genetic diversity retained within these populations. The fact that the EV population is regionally restricted and its small census size of herd-book cows explain its high level of genomic inbreeding.

**Electronic supplementary material:**

The online version of this article (10.1186/s12711-017-0358-6) contains supplementary material, which is available to authorized users.

## Background

Domestication, breed formation and intensive selection have led to divergent cattle breeds that likely exhibit distinctive genomic signatures of selection. Over recent years, molecular tools have contributed to a better understanding of domestication and have identified a growing list of genes involved in adaptation [1]. Numerous studies using various methods and types of molecular markers to characterize genetic diversity within and between breeds have been published [2]. Genome-wide single nucleotide polymorphism (SNP) data has pushed the characterization of genetic diversity in cattle breeds forward. Decker et al. [3] assessed the population structure of 134 cattle breeds using 50 K SNPs and identified three major groups of cattle: Asian indicine, Eurasian taurine, and African taurine. Comparing North American Brown Swiss, Jersey and Holstein bulls, Melka and Schenkel [4] found that the highest genetic differentiation was between Brown Swiss and Holstein bulls. A study that used multi-dimensional scaling to assess the population structure of Italian Brown, Italian Holstein, Piedmontese, Marchigiana and Italian Pezzata Rossa bulls showed that these five populations were separated from each other, with the Italian Brown showing a small group of outliers [5]. In a French study, French Holstein, Normande and Montbéliarde bulls were assigned to isolated clusters, whereas the population-specific average *F*_ST_ was highest for Holstein [6]. Another study that investigated population structure, selection signatures and demographic history in cattle was published by Oroczo-ter Wengel et al. [7].

Genome-wide SNP data allow the characterization of runs of homozygosity (ROH) which can quantify the extent of inbreeding in diploid individuals [8]. Several studies in cattle [9–13] showed that long and uninterrupted ROH were suitable to estimate genomic inbreeding coefficients. Knowledge of the ROH provides new possibilities to manage inbreeding in livestock species and could be used for optimal allocation of resources and maintenance of genetic variation in intensely selected bovine breeds [14]. Furthermore, ROH can be used to analyze inbreeding depression in cattle populations for which there is no reliable ancestry information [15–17].

The availability of genome-wide SNPs has led to the development of several methods for the detection of genomic regions that have undergone selection [18, 19]. Numerous studies using different methods for such analyses have been reported for cattle [5, 20–31]. Gutiérrez-Gil et al. [32] reviewed 21 studies and reported 1049 signatures of selection across 37 European cattle breeds. They highlighted private regions that were specific to single breeds, which may contain genes that are involved in the occurrence of unique phenotypic characteristics of such breeds. Randhawa et al. [33] performed a meta-analysis of 56 studies on signatures of selection that represented more than 70,000 animals from 90 bovine breeds. These authors reported hotspots of signatures of selection in the bovine genome, and identified regions under selection that were common to multiple breeds, some occurring in regions that contain single genes of known major effects and others that cover genes known to influence polygenic traits.

Genetic diversity is an intrinsic factor that influences the adaptive capacity and resilience of populations [34]. The objective of our study was to assess population structure using 27,612 autosomal SNPs in nine Swiss dairy cattle populations including Brown Swiss (BS), Braunvieh (BV), Original Braunvieh (OB), Holstein (HO), Red Holstein (RH), Swiss Fleckvieh (SF), Simmental (SI), Eringer (ER) and Evolèner (EV). In addition, we derived ROH and compared marker-based measures of inbreeding with pedigree-based inbreeding coefficients. For the derivation of potential signatures of selection, we calculated the *d*_*i*_ statistic [35], which is a function of pairwise *F*_ST_ values [36] between population *i* and the remaining populations to highlight potential loci that lead to differentiation between these populations. With the introduction of artificial insemination in the 1960’s, the OB population was introgressed with BS individuals from North America resulting in the current BV population [37], while genetic material of both RH and HO breeds were introduced into the SI population, resulting in the SF population [38]. Our findings help to improve our understanding of the genetic background of Swiss dairy cattle populations and enable the identification of differences between the original Swiss breeds (i.e. OB, SI, ER and EV) and those breeds that are now characterized by larger populations (i.e. BS, BV, HO, RH and SF).

## Methods

### Data and data preparation

The data analyzed consisted of 9214 bulls from nine Swiss cattle populations (see Additional file 1: Table S1), which were genotyped with Illumina Bovine 50k v1 or v2 SNP BeadChips (BS, BV, OB, HO, RH, SF, SI), Illumina 50k iSelect (ER) or Illumina Bovine 777k BeadChip (EV). In spite of the differences in SNP content between these BeadChips, 46,146 autosomal SNPs were common to the four genotyping arrays and these were used for quality control with PLINK 1.9 [39, 40]. In a first step, SNPs were filtered based on their calling rate (–geno 0.1) and 41,131 SNPs fulfilled this criterion. Second, for each population separately, SNPs with a minor allele frequency (–maf) lower than 1% or SNPs deviating from Hardy–Weinberg equilibrium (–hwe 0.0001) were removed. After this filtering step for all nine populations, 27,612 common SNPs were available for further analyses (see Additional file 2: Table S2).

### Population structure

We used various parameters to characterize population structure and genetic diversity. When not specified, these were obtained from PLINK 1.9 [39, 40]. The proportion of observed heterozygosity was estimated from the observed homozygosity (–het) as: 1 − number of observed homozygous loci/number of non missing loci. Genomic relationships represented by the genome-wide proportions of shared identical-by-descent alleles were derived for each pair of samples using the –genome option. Multi-dimensional scaling (MDS) of pairwise genetic distances was used to identify relationships between populations (–cluster –mds-plot 2). Pairwise *F*_ST_ values between the nine cattle populations were calculated using the SNP and Variation Suite v8 (Golden Helix, Inc., Bozeman, MT, www.goldenhelix.com). A graphical representation of the phylogenetic relationships between the nine populations was obtained by using the commonly applied neighbor-joining (NJ) method, as implemented in the program SPLITSTREE4 [41]. We used the program ADMIXTURE [42] to determine the optimal number of k clusters, and to characterize individuals in terms of these clusters. Due to the available relationship structures (e.g. half-sib structures) and differences in sample size, it was not possible to perform an ADMIXTURE analysis for the full sample set. Thus, we randomly resampled 50 individuals from each of the BS, BV, OB, HO, RH, SF and SI populations, while considering all 57 EV and ER animals, which resulted in 407 individuals. We used the software DISTRUCT [43] to draw a graphical representation of each cluster assignment by increasing k from 2 to 10.

### Genomic inbreeding

Genomic inbreeding coefficients for the 9214 bulls were derived by using the PLINK 1.9 [39, 40] option –het (*F*_HOM_ = [number of observed homozygous loci − number of expected homozygous loci]/[number of non-missing loci − number of expected homozygous loci]) and by using the option –homozyg (*F*_ROH_) with the following non-default settings: at least 50 SNPs to define a ROH (calculated according to Purfield et al. [9]), minimum SNP density set to 1 per 100 kb (average density in our data was 1 SNP every 90.1 kb), with a maximum gap length of 1800 kb (the maximum gap length in our data was 1737.1 kb). A window was considered as a ROH, if there were no heterozygous loci and no more than two missing genotypes for this region.

Inbreeding coefficients (*F*_ROH_) for each breed were calculated according to McQuillan et al. [44]:

$$F_{\text{ROH}} = \sum \frac{{L_{ROH} }}{{L_{AUTO} }},$$

where *L*_*AUTO*_ is the length of the autosomal genome that spans SNP positions [2,497,129 kb in the current study; (see Additional file 2: Table S2)]. Pedigree-based inbreeding coefficients (*F*_PED_) were derived for the 9214 bulls based on pedigree data with the Software CFC [45]. *F*_PED_ and *F*_ROH_ were compared using linear regression and Pearson’s correlation coefficients, across all animals or only for animals with at least 95% known ancestors across the last five generations (pedigree completeness index, PCI_5G_ ≥ 0.95).

### Selection signatures

The filtered data representing 9214 sires and 27,612 SNPs were used for the detection of signatures of selection. Due to the very limited number of samples and the close mutual relationships between the sampled individuals [46], ER and EV were pooled to infer signatures of selection.

Wright’s *F*_ST_ values were calculated for all 28 pairs of populations using the plink command –fst. Then, *d*_*i*_*SNP*_ values were calculated for each SNP and population as: $d_{i\_SNP} = \sum\nolimits_{j \ne i} {\frac{{F_{\text{ST}}^{ij} - E\left[ {F_{\text{ST}}^{ij} } \right]}}{{sd\left[ {F_{\text{ST}}^{ij} } \right]}}}$, where $E\left[ {F_{\text{ST}}^{ij} } \right]$ and $sd\left[ {F_{\text{ST}}^{ij} } \right]$ denote the expected value and standard deviation of *F*_ST_ between populations *i* and *j* calculated based on all 27,612 SNPs, as proposed by Akey et al. [35].

The *d*_*i*_ values were averaged for SNPs in 2435 non-overlapping 1-Mb windows. Windows with less than four SNPs were discarded, which resulted on an average of 11.27 SNPs per window (maximum = 26 SNPs). If the average *d*_*i*_ value of a window exceeded the 99th percentile of the empirical distribution of *d*_*i*_, it was considered significant, resulting in 25 windows as putative signatures of selection for each breed.

All genes that were present within 1 Mb up- or downstream of the middle position of the 25 population-specific significant windows were identified with the NCBI MAPVIEWER (http://www.ncbi.nlm.nih.gov/projects/mapview/; NCBI annotation release 104). Knowledge of population-specific characteristics (see Additional file 1: Table S1) and insights from the literature were combined to select the candidate genes that are reported in this study.

## Results

### Population structure

The mean within-population genomic relationship ranged from 0.044 (SF) to 0.155 (BS), whereas the mean observed heterozygosity ranged from 0.357 (BS) to 0.399 (SF) (Table 1 and see Additional file 3: Figures S1 and S2). Within BV, BS and SI, some highly related pairs of animals were found (see Additional file 4: Figure S3).

**Table 1:** Number of sires, average genomic relationship and average observed heterozygosity in nine Swiss dairy cattle populations

Population	Number of sires	Mean genomic relationship^a^ (± SD) (p < 2.2e−16)*	Mean observed heterozygosity (± SD) (p < 2.2e−16)*
BS	281	0.155 (± 0.067)^A^	0.357 (± 0.015)^A^
BV	3386	0.113 (± 0.053)^B^	0.364 (± 0.016)^B^
OB	167	0.082 (± 0.064)^C^	0.383 (± 0.012)^C^
HO	2568	0.083 (± 0.057)^D^	0.377 (± 0.014)^D^
RH	1960	0.060 (± 0.052)^E^	0.385 (± 0.015)^E^
SF	547	0.044 (± 0.051)^F^	0.399 (± 0.013)^F^
SI	248	0.124 (± 0.063)^G^	0.367 (± 0.012)^B^
ER	36	0.066 (± 0.024)^E^	0.381 (± 0.007)^C,D,E^
EV	21	0.091 (± 0.120)^C,D^	0.360 (± 0.024)^A,B^

*BS* Brown Swiss, *BV* Braunvieh, *OB* Original Braunvieh, *HO* Holstein, *RH* Red Holstein, *SF* Swiss Fleckvieh, *SI* Simmental, *ER* Eringer, *EV* Evolèner

* *p* value, Kruskal–Wallis test, R: kruskal.test()

^a^Pairwise genomic relationships were calculated for all individuals from all populations together

^A,B,C,D,E,F,G^Different letters indicate significant Bonferroni-adjusted differences between breeds, as assessed with the R-package DUNN.TEST

**Fig. 1:**
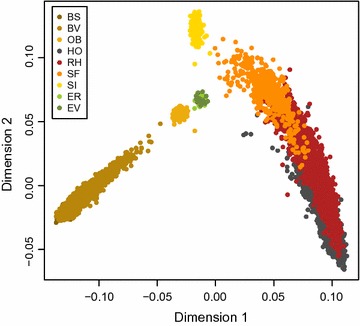
MDS-plot of dimension 1 versus dimension 2

Multi-dimensional scaling (MDS) of pairwise genetic distances was used to visualize relationships between the 9214 bulls. Plotting the first dimension versus the second dimension revealed five distinct clusters (Fig. 1). The original Swiss cattle populations OB, SI, ER, and EV are clearly separated from the larger, more common Swiss cattle populations and are positioned in-between the clusters of BV, BS and SF, RH, HO. An ADMIXTURE analysis (see Additional file 5: Figure S4) similarly demonstrated that OB, SI, ER and EV form distinct populations. In addition, these results further support the previously described genetic proximity between EV and ER, between BS and BV and between HO, RH and SF, respectively.

The *F*_ST_ values ranged from 0.007 (BS/BV) to 0.156 (BS/HO) in the investigated populations (see Additional file 6: Table S3). The NJ tree (see Additional file 7: Figure S5) illustrates the phylogenetic relationships between the nine populations based on *F*_ST_ distances. These results are in agreement with the clustering from the MDS plot (Fig. 1) and with the results from the ADMIXTURE analysis (see Additional file 5: Figure S4).

### ROH and genomic inbreeding

For 34 bulls, we detected no ROH segments (Table 2). The proportion of animals without a single ROH was highest in SF (2.9%), followed by OB (1.8%). In total, 135,640 ROH segments were identified across the nine populations and all the individuals. The average number of ROH per animal was largest for BS (21.0) and smallest for SF (7.1). The average sum of the lengths of ROH per animal ranged from 66.2 Mb (ER) to 226.4 Mb (BS). Additional file 8: Figure S6 represents the number and total length of ROH for the nine populations.

**Table 2:** Number of sires with and without identified runs of homozygosity (ROH), total number of ROH per population, average number of ROH per population and average sum of the lengths of ROH for each of the nine Swiss dairy cattle populations

Population	Number of sires without ROH	Number of sires with detected ROH	Total number of ROH	Avg. number of ROH segments (min–max) (p < 2.2e−16)*	Avg. sum of the lengths of ROH segments (Mb) (min–max) (p < 2.2e−16)*
BS	0	281	5892	21.0 (6–38)^A^	226.4 (39.2–505.1)^A^
BV	7	3379	62,783	18.6 (1–38)^B^	184.6 (3.7–638.3)^B^
OB	3	164	1382	8.4 (1–27)^C^	73.7 (4.9–234.6)^C^
HO	5	2563	36,498	14.2 (2–32)^D^	145.2 (8.2–696.3)^D^
RH	3	1957	21,979	11.2 (1–30)^E^	112.1 (4.0–460.8)^E^
SF	16	531	3772	7.1 (1–21)^C^	75.6 (3.4–273.5)^C^
SI	0	248	2703	10.9 (2–26)^E^	96.6 (13.2–299.8)^F^
ER	0	36	305	8.5 (2–15)^C,E^	66.2 (9.6–171.5)^C, F^
EV	0	21	326	15.5 (6–30)^B,D^	185.7 (35.0–371.3)^A, B, D^
Total	34	9180	135,640		

*BS* Brown Swiss, *BV* Braunvieh, *OB* Original Braunvieh, *HO* Holstein, *RH* Red Holstein, *SF* Swiss Fleckvieh, *SI* Simmental, *ER* Eringer, *EV* Evolèner

* *p* value, Kruskal–Wallis test, R: kruskal.test()

^A,B,C,D,E,F^Different letters indicate significant Bonferroni-adjusted differences between breeds, as assessed in the R-package DUNN.TEST

Long ROH are expected in inbred animals with recent common ancestors whereas short ROH reflect more distant common ancestors. Five- to 10-Mb long ROH were the most frequent in all populations, ranging from 43.2% (BS) to 47.5% (SI) (see Additional file 9: Figure S7). Comparison across the nine populations showed that, for ER, OB and SI, the highest proportion of ROH was for 1- to 5-Mb long ROH (34.8% in ER, 29.5% in OB and 29.2% in SI) and the lowest proportion for 10- to 15-Mb ROH (11.0% in SI, 11.5% in ER and 12.8% in OB).

The number of ROH per chromosome tended to increase with increasing chromosome length, with the largest numbers of ROH observed on BTA1 and 6 and the smallest on BTA27 and 5 (see Additional file 10: Table S4).

Overall, although available pedigree information for ER and EV was incomplete compared to the complete pedigree data for the seven other populations, ER and EV had the lowest average PCI and consequently, the lowest average pedigree inbreeding (*F*_PED_) estimates (Table 3). Average *F*_PED_ was highest for BS (7.1%), followed by BV (5.9%) and HO (5.7%) and average genomic inbreeding (*F*_HOM_, *F*_ROH_ and $F_{{{\text{ROH}} > 5Mb}}$) was highest for BS, BV and EV. Average *F*_HOM_ and *F*_ROH_ were higher than *F*_PED_ for all populations. Genomic inbreeding was slightly lower or equal to *F*_PED_ in HO, RH and SF when inbreeding was defined for long ROH using $F_{{{\text{ROH}} > 5Mb}}$.

**Table 3:** Number of sires, pedigree completeness index for five generations (PCI_5G_), average pedigree inbreeding (*F*_PED_) and average genomic inbreeding (*F*_HOM_, *F*_ROH_ and $F_{{{\text{ROH}} > 5{\text{Mb}}}}$) for nine Swiss dairy cattle populations

Population	Number of sires	Avg. PCI_5G_	Avg. *F*_PED_ (± SD) (p < 2.2e−16)*	Avg. *F*_HOM_ (± SD) (p < 2.2e−16)*	Avg. *F*_ROH_ (± SD) (p < 2.2e−16)*	Avg. $F_{{{\text{ROH}} > 5Mb}}$ (± SD) (p < 2.2e−16)*
BS	281	0.993	0.071 (± 0.028)^A^	0.115 (± 0.037)^A^	0.091 (± 0.029)^A^	0.084 (± 0.029)^A^
BV	3386	0.995	0.059 (± 0.023)^B^	0.100 (± 0.039)^B^	0.074 (± 0.028)^B^	0.067 (± 0.027)^B^
OB	167	0.992	0.023 (± 0.017)^C^	0.052 (± 0.029)^C^	0.029 (± 0.017)^C^	0.025 (± 0.016)^C^
HO	2568	0.991	0.057 (± 0.023)^D^	0.066 (± 0.035)^D^	0.058 (± 0.025)^D^	0.053 (± 0.025)^D^
RH	1960	0.993	0.042 (± 0.022)^E^	0.047 (± 0.037)^C^	0.045 (± 0.023)^E^	0.041 (± 0.023)^E^
SF	547	0.992	0.027 (± 0.020)^C^	0.012 (± 0.033)^E^	0.029 (± 0.021)^C^	0.027 (± 0.021)^B^
SI	248	0.991	0.028 (± 0.024)^C^	0.092 (± 0.030)^B^	0.039 (± 0.023)^F^	0.033 (± 0.022)^F^
ER	36	0.808	0.015 (± 0.012)^C^	0.056 (± 0.018)^C,D^	0.027 (± 0.014)^C,F^	0.022 (± 0.013)^B,F^
EV	21	0.358	0.012 (± 0.026)^C^	0.109 (± 0.059)^A,B^	0.074 (± 0.042)^B,D^	0.070 (± 0.041)^A,B,D^

*BS* Brown Swiss, *BV* Braunvieh, *OB* Original Braunvieh, *HO* Holstein, *RH* Red Holstein, *SF* Swiss Fleckvieh, *SI* Simmental, *ER* Eringer, *EV* Evolèner

* *p* value, Kruskal–Wallis test, R: kruskal.test()

^A,B,C,D,E,F^Different letters indicate significant Bonferroni-adjusted differences between breeds, as assessed in the R-package DUNN.TEST

**Fig. 2:**
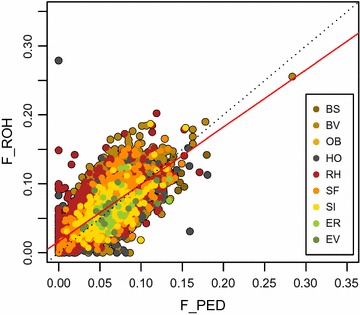
Regression of *F*_PED_ on *F*_ROH_ for all 9214 individuals. Multiple R-squared: 0.49. Red line is regression line (*F*_ROH_ = 0.016 + 0.830 * *F*_PED_)

Linear relationships between *F*_ROH_ and *F*_PED_ (Fig. 2) and between *F*_HOM_ and *F*_PED_ (see Additional file 11: Figure S8) were observed. Across all 9214 animals, the correlations between *F*_PED_ and *F*_ROH_ and between *F*_PED_ and *F*_HOM_ were significantly different from 0 with r equal to 0.70 (p < 2.2e−16) and 0.67 (p < 2.2e−16), respectively (see Additional file 12: Tables S5 and S6) and the correlation between *F*_PED_ and $F_{{{\text{ROH}} > 5Mb}}$ was equal to 0.69 (results not shown).

For 12 chromosomes (i.e. BTA3, 4, 6, 10, 11, 12, 13, 18, 19, 20, 22, and 29), at least 25% of the investigated animals within a population shared a ROH (see Additional file 13: Figure S9).

### Selection signatures

For each population, 25 windows were considered to represent putative signatures of selection, which resulted in 200 significant windows across the eight investigated groups of populations, i.e. when ER and EV populations were considered in the same group (see Additional file 14: Figure S10, Additional file 15: Table S7). Among these windows, 66 (33%) were significant but private to only one population. The remaining 134 (67%) windows were significant in two or more populations, with one extreme window on BTA14 showing significant *d*_*i*_ values in all populations (Table 4 and see Additional file 15: Table S7). Across all groups and autosomes, BTA5 hosted the largest number of significant windows (N = 33), followed by BTA6 (N = 32), BTA18 (N = 16) and BTA20 (N = 15). Almost 50% of the significant windows are located on these four autosomes.

**Table 4:** Genomic coordinates (chromosome and start- and stop-position) of the 32 windows with *d*_*i*_ higher than 10, the corresponding population (in brackets, other populations for which the window was significant) and their candidate genes

BTA	Start position	End position	*d*_*i*_	Population (other populations)	Candidate genes	Associated trait	References
4	53,358,285	55,358,285	13.424	ER/EV			
4	78,602,192	80,602,192	10.036	ER/EV	*INHBA*	Feed intake	[71]
5	16,275,624	18,275,624	10.760	SF (BV, OB, ER/EV)	*KITLG*		[80]
5	46,225,174	48,225,174	11.852	RH (HO, SF, ER/EV)			
5	46,225,174	48,225,174	11.511	HO (RH, SF, ER/EV)			
5	55,558,224	57,558,224	13.108	SI (RH, SF)	*STAT6*	Growth efficiency, carcass traits	[81]
5	60,520,962	62,520,962	12.197	SI			
5	75,148,807	77,148,807	10.435	SI (BV, BS)	*SYT10*	Longevity	[82]
5	76,432,958	78,432,958	10.735	BS (BV, OB)	*SYT10*	Longevity	[82]
6	32,407,621	34,407,621	11.691	BV (BS)			
6	69,255,003	71,255,003	13.069	SI (BV, BS, HO, SF)	*KIT*	White spotting	[80]
6	77,416,656	79,416,656	12.928	HO (BS, BV, RH, SF, BS, BV)			
6	77,416,656	79,416,656	11.932	RH (BS, BV, HO, SF,)			
11	65,512,895	67,512,895	12.856	OB	*PROKR1*	Fertility	[48]
11	67,608,705	69,608,705	17.985	OB (BV, SF, SI, ER/EV)	*CAPN14*, *CAPN13*, *LBH*, *LCLAT1*, *GFPT1*, *EHD3*, *GMCL1*, *PCBP1*	Fertility, meat quality	[48, 75, 76]
11	67,608,705	69,608,705	11.660	SI (BV, OB, SF, ER/EV)			
14	23,392,111	25,392,111	18.647	RH (BS, BV, OB, HO, SF, SI, ER/EV)	*PLAG1*, *CHCHD7*, *LYN*, *TGS1*	Stature, fertility	[69, 70]
14	23,392,111	25,392,111	15.509	SF (BS, BV, OB, HO, RH, SI, ER/EV)			
14	23,392,111	25,392,111	15.050	HO (BS, BV, OB, RH, SF, SI, ER/EV)			
14	23,392,111	25,392,111	10.466	OB (BS, BV, HO, RH, SF, SI, ER/EV)			
16	24,736,714	26,736,714	15.161	BV (BS, OB, RH, SF, SI)	*HLX*	Feed efficiency	[83]
16	24,736,714	26,736,714	14.335	BS (BV, OB, RH, SF, SI)			
16	26,450,435	28,450,435	10.846	BS (BV, OB)	*TLR5*, *CAPN8*, *CAPN2*	Disease resistance, meat quality	[75, 84–86]
18	13,233,845	15,233,845	25.720	HO (OB, RH, SF, SI)	*MC1R*, *SLC7A5*, *CDH15*, *GALNS*, *FANCA*	Coat color	[31–33, 87]
18	13,233,845	15,233,845	24.272	RH (OB, HO, SF, SI)			
18	13,233,845	15,233,845	10.451	SF (OB, HO, RH, SI)			
18	14,373,569	16,373,569	13.715	RH (HO, SF)	*FANCA*		[87]
20	29,194,786	31,194,786	14.835	HO (RH)	*MRPS30*, *FGF10*	Milk yield. Protein percentage	[88, 89]
20	43,561,002	45,561,002	10.593	HO (RH)			
20	46,566,649	48,566,649	12.743	HO (RH, SF, SI)	*CDH9*	Clinical mastitis	[90]
20	46,566,649	48,566,649	10.759	RH (HO, SF, SI)	*CDH9*	Clinical mastitis	[90]
26	21,528,510	23,528,510	13.360	ER/EV (OB)	*FGF8*, *SCD*	Carcass quality, fertility, fatty acid composition	[72–74]

The 32 most prominent signatures of selection (e.g. windows with *d*_*i*_ higher than 10) are in Table 4. The window with the highest *d*_*i*_ (25.7) was on BTA18 (14.2 Mb) in HO (Table 4). The same window had the second highest *d*_*i*_ (24.3) in RH and was also significant in SF, SI and OB. The window with the third highest *d*_*i*_ (18.6) was on BTA14 (24.4 Mb) in RH. This latter window was significant in all populations and was previously reported to harbor signatures of selection (e.g. [31, 33, 47]).

The window with the fourth highest *d*_*i*_ was on BTA11 (68.6 Mb) in OB and was also significant in BV, SI, SF and ER/EV. It was previously reported in OB [48]. With a *d*_*i*_ of 12.9, the preceding window (i.e. on BTA11 between 65.5 and 67.5 Mb) was also significant in OB. A window with a significant *d*_*i*_ higher than 14 was found on BTA16 (between 24.7 and 26.7 Mb) in BV and BS and was also significant in OB, RH, SF and SI. In the same region, another window with a *d*_*i*_ higher than 10 was significant in BS (BTA16 between 26.5 and 28.5 Mb) and was also significant in BV and OB.

Three strong signatures of selection (i.e. *d*_*i*_ higher than 10) were observed for HO on BTA20, i.e. in windows spanning 29.2–31.2, 43.5–45.6 and 46.6–48.6 Mb. All three windows were also significant in RH whereas the third window was significant in SF and SI. In the top two significant windows that spanned 43.5–45.6 and 46.6–48.6 Mb on BTA20, we did not identify any candidate genes (Table 4 and see Additional file 15: Table S7). Remarkably, the proximal window (BTA20 between 30.5 and 32.5 Mb) that harbors the well-known *GHR* gene was not significant in HO but moderately significant in RH (*d*_*i*_ = 7.4) and in SF (*d*_*i*_ = 5.9) (see Additional file 15: Table S7).

For ER/EV, two windows with *d*_*i*_ of 13.4 were identified on BTA4 (between 53.4 and 55.4 Mb) and on BTA26 (between 21.5 and 23.5 Mb). The BTA26 window was also significant in OB, another local Swiss cattle population. Among the nine loci localized in the region of the significant window on BTA4, no obvious candidate gene was identified. A second signature of selection on BTA4 (between 78.6 and 80.6 Mb) was private to ER/EV.

In SI, a window with a *d*_*i*_ of 13.1 was observed on BTA6 (between 69.3 and 71.3 Mb) and was also significant in BS, BV, HO and SF. The *KIT* gene (BTA6: 71.8–71.9 Mb) is located near this region. Based on the filtered dataset, the available SNP density in this region was very limited. Similarly, the window on BTA5 (between 16.3 and 18.3 Mb) was significant in five populations (BV, OB, SF and ER/EV). On BTA5, two additional windows (between 55.6 and 57.6 Mb and between 60.5 and 62.5 Mb) were highly significant in the sampled SI individuals, with the first window (55.6–57.6 Mb) being also significant in the RH and SF populations. Two other windows on BTA5 were significant: i.e. the window between 75.1 and 77.1 Mb in SI, BS and BV and the window between 76.4 and 78.4 Mb in BS, BV and OB.

Additional windows with a *d*_*i*_ higher than 10 were detected on BTA5 (between 46.2 and 48.2 Mb and between 60.5 and 62.5 Mb) and BTA6 (between 32.4 and 34.4 Mb and between 77.4 and 79.4 Mb) but no candidate genes were identified in any of these windows.

## Discussion

### Population structure

We assessed the population structure of nine Swiss cattle populations by using 27,612 autosomal SNPs and showed that genetic diversity was highest in SF and lowest in BS. Based on *F*_ST_, BS and HO were more differentiated (0.156) than all other pairs of populations (see Additional file 6: Table S3), which agrees with the results reported by Melka and Schenkel [4]. The genetic differentiation was lowest in BS and BV (0.007), followed by HO and RH (0.016), RH and SF (0.025), ER and EV (0.048), HO and SF (0.052). These findings are in concordance with the MDS plot (Fig. 1), which shows five distinct clusters: (1) BS and BV, (2) OB, (3) ER and EV, (4) SI, and (5) SF, RH and HO, and with the results from the ADMIXTURE analysis. The original Swiss cattle populations OB, SI, ER, and EV that are currently less common are clearly separated from the more common cattle populations. Combining the MDS and ADMIXTURE results with the *F*_ST_ values of 0.111 for BS and OB and 0.094 for BV and OB, the clear separation between OB and BS and BV is obvious. The OB population represents the original population of Brown cattle without the influence of the recently introgressed BS individuals. However, OB is ancestral to the BS population, which was formed in the USA from animals that were obtained in Switzerland between 1869 and 1910 [37]. According to Porter et al. [49], the BS breed was founded based on 167 of these imported individuals. In the USA, this BS founder population was improved with a specific selection focus on milk yield. Introgression of BS back into OB began in Switzerland in the 1960s, which coincided with the introduction of artificial insemination and subsequently led to the BV population. The use of imported BS sires in BV is still common and thus leads to BV animals with various levels of BS genes [37, 50]. SF is a crossbreed between SI and RH (see Additional file 5: Figure S4). Based on the *F*_ST_ values, SF is more distant from SI (0.072) than from RH (0.024). This is also apparent in the MDS plot, where SF is more distant from SI than from RH. The comparison of within-population diversity, which was quantified either from genomic relationships or from observed heterozygosity, showed that the two extreme populations were SF and BS with the highest diversity observed in SF and the lowest diversity in BS. This is not surprising for two reasons: SF is well known as a composite population of SI and RH [38] and, as indicated above, BS can be traced back to a few OB founder animals that were subsequently strongly selected for milk production. The considerable loss of genetic diversity within the BS population was previously reported based on pedigree information [37].

### Genomic inbreeding

In recent years, several studies have investigated ROH in cattle. Purfield et al. [9] found similar correlations between *F*_ROH_ and *F*_PED_ using both 50k and HD SNP data and thus, they concluded that 50k SNP data are sufficient to identify ROH and to estimate genomic inbreeding. Because the parameters used to detect ROH vary among analyses, it is not easy to compare results from different ROH studies. The setting of the parameters used to derive ROH is crucial to account for the effects of SNP density correctly. Using the PLINK 1.9 [39, 40] default parameter of 100 consecutive SNPs to call a ROH, would not have identified any ROH less than 5 Mb in our data (results not shown). Therefore, the minimum number of SNPs to identify ROH should be defined according to the available SNP density. One such approach was proposed by Lencz et al. [51] and applied by Purfield et al. [9]. Their conclusions were supported by the recent study of Rodriguez-Ramilo and Fernandez [52] who showed that the four parameters, minimum length, minimum number of SNPs, minimum SNP density and maximum distance between two adjacent SNPs, each influence the identification of ROH and therefore the estimation of *F*_ROH_. In our study, we defined ROH as tracts of homozygous SNPs that spanned a minimum of 50 consecutive loci, in regions with a minimum density of one SNP every 100 kb, a maximum gap length of 1800 kb, while allowing up to two missing genotypes per window but no heterozygous loci. Most ROH were observed in the length class of 5 to 10 Mb (see Additional file 9: Figure S7). This contrasts with the findings of Marras et al. [12] who reported that the 1- to 2-Mb long ROH were the most frequent in all populations, ranging from ~ 50% in Italian Brown to ~ 80% in Piedmontese. The difference between these results is mainly explained by the different minimum number of SNPs used to define a ROH. In our data, frequencies higher than 28% were observed for the shortest ROH length class (1 to 5 Mb) in the three local populations OB, SI and ER. This could be due to “old inbreeding” resulting from previous bottlenecks that occurred when the breed was created during the second half of the nineteenth century. However, the frequencies of the longest ROH, i.e. length classes of 25 to 30 Mb and more than 30 Mb were highest in EV, which indicates recent inbreeding. This is not surprising because this population is specific to a given region and its census size is less than 200 registered herd-book cows (see Additional file 1: Table S1).

Mean levels of *F*_ROH_ ranged from 0.027 (ER) to 0.091 (BS). The results for BS (0.091) and BV (0.074) are consistent with their low levels of genetic diversity. In contrast, the three original Swiss cattle populations ER (*F*_ROH_: 0.027), OB (*F*_ROH_: 0.029), and SI (*F*_ROH_: 0.039) had lower levels of inbreeding. Natural service is still commonly used in these three populations (see Additional file 1: Table S1), which requires the use of more sires at each generation than artificial insemination. This is considered as the major reason for the remarkably low levels of genomic inbreeding within these populations, although they have been closed populations for a long time.

*F*_ROH_ directly reflects the level of homozygosity and is not influenced by allele frequencies, unlike *F*_HOM_, which depends on allele frequencies and thus on sampling [53]. *F*_HOM_ can even be negative for some individuals, which indicates that they are less inbred than the average population [54]. Nevertheless, since *F*_HOM_ is a single point approach, it does not rely on the availability of SNP positions [55]. For ROH analyses, the knowledge of SNP positions is an important prerequisite. The fact that *F*_ROH_ does not depend on the sampling procedure is a great advantage since ROH can be identified for every single individual. Furthermore, with *F*_ROH_, recent and ancient inbreeding can be distinguished [8, 56].

Our results clearly showed a linear relationship between *F*_ROH_ and *F*_PED_ and between *F*_HOM_ and *F*_PED_ (Fig. 2 and see Additional file 11: Figure S8, Additional file 12: Tables S5 and S6). The correlations between *F*_ROH_ and *F*_PED_ presented here are in concordance with published results in cattle [9, 12] and other species with similar pedigree completeness such as goats [57] and horses [58].

Several of the regions in which we identified ROH that were common to at least 25% of the animals (see Additional file 13: Figure S9) confirm previously reported data. For example, on BTA6 we detected a ROH that was present in at least 25% of the investigated BS and BV bulls and located in the same region (~ 91 Mb) where Schwarzenbacher [14] claimed that up to 50% of Brown Swiss bulls carried a ROH. A possible explanation for the obvious inbreeding in this region is that it harbors several QTL for economically relevant traits in cattle such as protein yield [59], clinical mastitis [60–62], milking speed [63] and udder traits [64, 65]. On BTA13, more than 30% of the BS bulls and 25% of the BV bulls had a ROH in the region between 30 and 40 Mb, which agrees with the studies of Minozzi et al. [66] who detected SNPs in this region (~ 30.5 Mb) that were significantly associated with days to first service in Holstein and of Stella et al. [23] who reported signatures of selection on this chromosome at ~ 33.0 Mb in dairy breeds. On BTA19, we found that up to 35% of the BS bulls had a ROH between 45 and 50 Mb, which is concordant with the high level of genetic differentiation at ~ 46 Mb reported by the Bovine HapMap Consortium [22]. Furthermore, in a Braunvieh population, Rothammer et al. [48] detected a signature of selection in the region between ~ 47 and 51 Mb on BTA19, which harbours the *GH1* gene, a potential candidate gene for dairy cattle production traits. On BTA10, we showed that up to 30% of the HO bulls had a ROH between 50 and 60 Mb. Previously, in a study on German Holstein, Kühn et al. [67] identified putative QTL for somatic cell content and non-return rate at 90 days (paternal effect) on BTA10 between 34.7 and 56.9 Mb. Furthermore, based on integrated haplotype scoring (iHs) on Holstein data, the Bovine HapMap Consortium [22] reported recent positive selection at ~ 53 Mb on BTA10. Finally, on BTA18, up to 30% of the RH bulls investigated in our study had a ROH between 10 and 20 Mb, which is a region that includes the well-known *MC1R* gene and where composite signatures of selection were detected in several breeds (e.g. [32]).

### Signatures of selection

Various studies on signatures of selection in cattle using genome-wide SNPs have been published and for dairy cattle populations such as Holstein, Red Holstein and Brown Swiss, major signatures of selection have been described (e.g. [31]). In their study, Rothammer et al. [48] included local cattle populations and derived signatures of selection for the OB population based on 50k genotypes from 35 individuals. Signatures of selection were also identified in the SI populations by Fan et al. [29] and Zhao et al. [31], among others. To our knowledge, our study is the first one to consider data from ER and EV populations, which are well-known to have a long selection history with emphasis on milk, meat and fighting ability traits [49, 68]. Thus, our study that includes samples from OB, SI, and ER/EV breeds investigates for the first time signatures of selection for dairy cattle populations in Switzerland.

Genes known to be linked to strong signatures of selection in cattle such as *KIT*, *MC1R*, *ABCG2*, *LCORL/NCAPG* and *PLAG1* [33] are fully supported by our data. These signatures of selection were significant in many of the analyzed breeds with the most extreme signal around *PLAG1* being significant in all eight population groups. The pleiotropic nature of this region [69] and a potentially interesting mutation for bovine stature [70] are understood to be major drivers that underlie the strong signature of selection that was observed among all Swiss dairy populations.

Selection at the *POLL* locus, the *MSTN* and *DGAT1* genes, and the genes from the casein cluster have not left any recognizable signatures of selection among the investigated populations. Based on phenotypic evidence, it is presumed that the region that includes the casein cluster is under selection but that it has not yet reached fixation in any of the Swiss populations. We did not detect any signature of selection in the *DGAT1* gene, possibly because of the low SNP density in this region of BTA14. Since the Swiss dairy cattle populations are historically horned and not influenced by any double-muscled breed, the lack of signatures of selection around the *POLL* locus and the *MSTN* gene was not surprising.

The common ancestries between BS, BV and OB, between HO, RH and SF and between SF and SI are well known and were previously described [37, 38]. Thus, the detection of private signatures of selection in the original Swiss SI, OB and ER/EV populations was of major interest for this study. On BTA4, two prominent signatures of selection (between 53.4 and 55.4 Mb and between 78.6 and 80.6 Mb) were detected only in the ER/EV population. Although we identified no obvious candidate gene in the BTA4 window between 53.4 and 55.4 Mb based either on the literature or functional evidence, a meta-assembly of signatures of selection suggested that this region is under selection in European breeds [33]. For the second BTA4 window between 78.6 and 80.6 Mb, the *INHBA* gene represents a relevant candidate gene since it is involved in feed intake in Angus cattle [71]. We detected a strong signature of selection in ER/EV that was also significant in OB in the BTA26 region between 21.5 and 23.5 Mb, which harbors genes such as *FGF8* and *SCD*. These two candidate genes are known to influence carcass quality, fatty acid composition of meat and milk and fertility traits [72–74]. Due to their pleiotropic effects on these economically important traits, and to the strength of the signal, further investigations on the functional consequences of this signature of selection are required. Two windows on BTA11 (between 65.5 and 67.5 Mb and between 67.6 and 69.6 Mb) harbored highly significant signatures of selection in the OB population. Previously, Rothammer et al. [48] assigned the most extended signature of selection for the OB population to this region and proposed genes associated with fertility as possible candidate genes. However, other genes such as *CAPN14*, *CAPN13*, *LBH* and *LCLAT1* genes that are located in this region and influence meat quality [75, 76] should also be considered as candidate genes. For the SI population, two windows on BTA5 (between 55.6 and 57.6 Mb and between 60.5 and 62.5 Mb) are particularly interesting. Between these two windows, another region that spans the *PMEL* and *GDF11* genes is characterized by a *d*_*i*_ higher than 20 in SI (results not shown) but it was omitted from the final derivation of signatures of selection, because it did not fulfill the minimal SNP density of four loci per window. Based on these results, it is suggested that the extended BTA5 region between 55.6 and 62.5 Mb played an important role in the differentiation of SI. Besides many other loci, we propose *STAT5*, *GDF11* and *PMEL* as potential drivers of the differentiation of SI from other cattle populations.

For several of the significant regions representing signatures of selection, we did not identify any candidate genes either due to the lack of genes with functional evidence in these regions (e.g. BTA4 between 53.4 and 55.4 Mb; Table 4) or to poor annotation. Zhao et al. [31] suggested that regions that do not appear to contain genes may play an important role in adaptation and may be elucidated in the future with an improved annotation of the bovine genome. Based on limited marker densities in our data and the finding of Kemper et al. [47] that response to selection is usually based on small changes in frequency at many loci, only loci with major effects due to strong selection could be detected here. The collection and analysis of thousands of phenotypes together with high-density genotypes may be necessary to disentangle the genetic basis of adaptation to the alpine environment of SI, OB and ER/EV populations.

## Conclusions

The original Swiss cattle populations OB, SI, ER and EV are genomically distinct from the more common dairy cattle populations. We report several private signatures of selection in regions that harbor genes such as *INHBA*, *STAT6*, *PROKR1*, *CAPN14*, *CAPN13*, *FGF8* and *SCD* for these original populations. The low levels of genomic inbreeding observed in OB, SI and ER might be explained by the continued use of natural service sires, which is likely the major reason for their remarkably high level of genetic diversity although these populations have been closed for a long time. The regional specificity and the small census size of herd-book cows in EV explain its high level of genomic inbreeding. Optimum genetic contribution selection [77–79] may be an option to avoid inbreeding in the more popular Swiss dairy cattle represented by larger populations and in which the proportion of artificial insemination is higher than 90%.

## Additional files

**Additional file 1: Table S1.** Number of genotyped bulls per population with their minimum and maximum birth year, as well as number of breeding animals per population, responsible breeding organization and breed-specific characteristics such as major use, breeding goal and proportion of artificial insemination.

**Additional file 2: Table S2.** Number of SNPs per chromosome, range covered by SNPs per chromosome and in total.

**Additional file 3: Figure S1.** Boxplots of genomic relationships. **Figure S2.** Boxplots of observed heterozygosity.

**Additional file 4: Figure S3.** Levelplot of pair-wise genomic relationship within and between populations.

**Additional file 5: Figure S4.** Distruct plot of the Admixture results for the nine Swiss cattle populations. Cross-validation error was lowest for k = 9 and 10, which indicates that k = 9 or 10 is the optimal number of clusters.

**Additional file 6: Table S3.** *F*_ST_-values with 95% confidence interval in brackets in the lower triangular part.

**Additional file 7: Figure S5.** Neighbour joining (NJ) tree based on *F*_ST_ distances.

**Additional file 8: Figure S6.** Relationship between the number of ROH and the total length of genome in ROH.

**Additional file 9: Figure S7.** Distribution of the number of ROH in different length classes and for each population.

**Additional file 10: Table S4.** Number of ROH per chromosome and population.

**Additional file 11: Figure S8.** Regression of *F*_PED_ on *F*_HOM_ for all 9214 individuals. Multiple R-squared: 0.45.

**Additional file 12: Table S5.** Correlations between *F*_PED_ and *F*_ROH_ and between *F*_PED_ and *F*_HOM_ including all animals. **Table S6.** Correlations between *F*_PED_ and *F*_ROH_ and between *F*_PED_ and *F*_HOM_ including only animals with PCI > 0.95.

**Additional file 13: Figure S9.** Proportion of animals with this SNP within a ROH.

**Additional file 14: Figure S10.**. Genomic distribution of the *d*_*i*_ statistic for all 1-Mb windows across all autosomes and the eight groups of populations. The dashed red line denotes the 99th percentile for each population group (BS: Brown Swiss, BV: Braunvieh, OB: Original Braunvieh; HO: Holstein, RH: Red Holstein, SF: Swiss Fleckvieh, SI: Simmental, ER/EV: Eringer and Evolèner).

**Additional file 15: Table S7.** Genomic coordinates (chromosome and start– and end–position) of all significant windows, the population for which *d*_*i*_ was calculated, and genes within this region.
