# Supplementary material for: Population structure and genomic inbreeding in nine Swiss dairy cattle populations
Source: Genet Sel Evol. 2017 Nov 7;49:83. doi: 10.1186/s12711-017-0358-6 (PMC5674839; doi:10.1186/s12711-017-0358-6)
Supplement: Supplementary file 1 — Additional file 1: Table S1. Number of genotyped bulls per population with their minimum and maximum birth year, as well as number of breeding animals per population, responsible breeding organization and breed-specific characteristics such as major use, breeding goal and proportion of artificial insemination. [file 12711_2017_358_MOESM1_ESM.docx]

Table S1 Number of genotyped bulls per population with their minimum and maximum birth year, as well as number of breeding animals per population, responsible breeding organization and breed specific characteristics such as major use, breeding goal and proportion of artificial insemination.

| **Population** | **Abbreviation** | **No. of bulls** | **Birth year** | | **Herdbook animals (2013)*** | | **Breeding organization** | **Use** | **Breeding goal** | **Proportion of artificial insemination** |
| --- | --- | --- | --- | --- | --- | --- | --- | --- | --- | --- |
|  |  |  | Min | max | Male >9 months | Female >5 months pregnant |  |  |  |  |
| Brown Swiss | BS | 281 | 1960 | 2013 | 507 | 183,773 | Braunvieh Schweiz:  www. Braunvieh.ch | Milk | 8500 kg milk, 138–150 cm (cow) | >90% |
| Braunvieh | BV | 3386 | 1973 | 2013 |  |  |  | Milk |  |  |
| Original Braunvieh | OB | 167 | 1972 | 2011 | 240 | 9832 |  | Milk and meat | 7500 kg milk, 135–145 cm (cow) | ~50% |
| Holstein | HO | 2568 | 1978 | 2013 | 744 | 104,603 | Holstein Switzerland: www.holstein.ch Swiss Herdbook: www.swissherdbook.ch | milk | 10,000 kg milk^2^, 150–155 cm (cow)^2^ | >90% |
| Red Holstein | RH | 1960 | 1985 | 2013 | 361 | 117,156 | Swiss Herdbook: www.swissherdbook.ch | Milk | 10,000 kg milk, 150–155 cm (cow) | >90% |
| Swiss Fleckvieh | SF | 547 | 1985 | 2013 | 187 | 67,594 |  | Milk | 8000 kg milk, 140–150 cm (cow) | >85% |
| Simmental | SI | 248 | 1985 | 2010 | 417 | 25,095 |  | Milk and meat | 7500 kg milk, 140–148 cm (cow) | >60% |
| Eringer | ER | 36 | 2000 | 2002 | 180 | 6527 | Swiss Federation of livestockfarming of the Hérens Breed: www.raceherens.ch | Milk, meat and fighting | 3500 kg milk, 120-130 cm (cow) | 50-60% |
| Evolèner | EV | 21 | 1992 | 2011 | 6 | 192 | Swiss Herdbook: swissherdbook.ch Evolèner Zuchtverein: www.evolener–zuchtverein.ch ProSpecieRara: www.prospecierara.ch | Milk and meat | 3500 kg milk, 115–125 cm (cow) | 50-60% |

*BLW – Förderung der Tierzucht, Rechnung 2014, Budget 2014.
