# Supplementary material for: Population structure and genomic inbreeding in nine Swiss dairy cattle populations
Source: Genet Sel Evol. 2017 Nov 7;49:83. doi: 10.1186/s12711-017-0358-6 (PMC5674839; doi:10.1186/s12711-017-0358-6)
Supplement: Supplementary file 2 — Additional file 2: Table S2. Number of SNPs per chromosome, range covered by SNPs per chromosome and in total. [file 12711_2017_358_MOESM2_ESM.docx]

Table S2 Number of SNP per chromosome, range covered by SNP per chromosome and in total.

| **BTA** | **Number of SNP** | **Range covered by SNP (kb)** |
| --- | --- | --- |
| 1 | 1820 | 158,094.120 |
| 2 | 1455 | 136,507.350 |
| 3 | 1359 | 120,807.283 |
| 4 | 1303 | 120,361.350 |
| 5 | 1095 | 121,078.748 |
| 6 | 1289 | 118,943.298 |
| 7 | 1228 | 112,384.068 |
| 8 | 1296 | 112,908.177 |
| 9 | 1114 | 105,464.416 |
| 10 | 1189 | 103,088.420 |
| 11 | 1125 | 107,136.657 |
| 12 | 915 | 90,829.292 |
| 13 | 966 | 83,835.601 |
| 14 | 1017 | 83,152.514 |
| 15 | 904 | 84,221.946 |
| 16 | 840 | 81,249.445 |
| 17 | 866 | 74,854.599 |
| 18 | 754 | 65,159.683 |
| 19 | 766 | 63,513.847 |
| 20 | 825 | 71,546.415 |
| 21 | 747 | 71,070.729 |
| 22 | 675 | 60,759.503 |
| 23 | 601 | 52,068.731 |
| 24 | 736 | 62,101.915 |
| 25 | 547 | 42,712.739 |
| 26 | 567 | 50,923.646 |
| 27 | 540 | 45,190.855 |
| 28 | 532 | 46,060.959 |
| 29 | 541 | 51,102.335 |
| total | 27,612 | 2,497,128.641 |
