# Supplementary material for: Population structure and genomic inbreeding in nine Swiss dairy cattle populations
Source: Genet Sel Evol. 2017 Nov 7;49:83. doi: 10.1186/s12711-017-0358-6 (PMC5674839; doi:10.1186/s12711-017-0358-6)
Supplement: Supplementary file 3 — Additional file 3: Figure S1. Boxplots of genomic relationships. Figure S2. Boxplots of observed heterozygosity. [file 12711_2017_358_MOESM3_ESM.docx]

Figure S1 Boxplots of genomic relationships.

Figure S2 Boxplots of observed heterozygosity.
