# Supplementary material for: Population structure and genomic inbreeding in nine Swiss dairy cattle populations
Source: Genet Sel Evol. 2017 Nov 7;49:83. doi: 10.1186/s12711-017-0358-6 (PMC5674839; doi:10.1186/s12711-017-0358-6)
Supplement: Supplementary file 4 — Additional file 4: Figure S3. Levelplot of pair-wise genomic relationship within and between populations. [file 12711_2017_358_MOESM4_ESM.docx]

Figure S3 Levelplot of pair-wise genomic relationship within and between populations.
