# Supplementary material for: Population structure and genomic inbreeding in nine Swiss dairy cattle populations
Source: Genet Sel Evol. 2017 Nov 7;49:83. doi: 10.1186/s12711-017-0358-6 (PMC5674839; doi:10.1186/s12711-017-0358-6)
Supplement: Supplementary file 5 — Additional file 5: Figure S4. Distruct plot of the Admixture results for the nine Swiss cattle populations. Cross-validation error was lowest for k = 9 and 10, which indicates that k = 9 or 10 is the optimal number of clusters. [file 12711_2017_358_MOESM5_ESM.docx]

Figure S4 Distruct plot of the Admixture results for the nine Swiss cattle populations. Cross-validation error was lowest for k = 9 and 10, which indicates that k = 9 or 10 is the optimal number of clusters.
