# Supplementary material for: Population structure and genomic inbreeding in nine Swiss dairy cattle populations
Source: Genet Sel Evol. 2017 Nov 7;49:83. doi: 10.1186/s12711-017-0358-6 (PMC5674839; doi:10.1186/s12711-017-0358-6)
Supplement: Supplementary file 6 — Additional file 6: Table S3. F ST-values with 95% confidence interval in brackets in the lower triangular part. [file 12711_2017_358_MOESM6_ESM.docx]

Table S3 F_ST_–values with 95% confidence interval in brackets in lower triangular

|  | **BS** | **BV** | **OB** | **HO** | **RH** | **SF** | **SI** | **ER** | **EV** |
| --- | --- | --- | --- | --- | --- | --- | --- | --- | --- |
| **BS** | 0.0000 | 0.0067 | 0.1108 | 0.1563 | 0.1427 | 0.1227 | 0.1495 | 0.1348 | 0.1528 |
| **BV** | 0.0067  (±0.0001) | 0.0000 | 0.0935 | 0.1453 | 0.1327 | 0.1128 | 0.1332 | 0.1173 | 0.1348 |
| **OB** | 0.1108  (±0.0017) | 0.0935  (±0.0015) | 0.0000 | 0.1193 | 0.1049 | 0.0816 | 0.1033 | 0.0855 | 0.1023 |
| **HO** | 0.1563  (±0.0022) | 0.1453  (±0.0021) | 0.1193  (±0.0019) | 0.0000 | 0.0164 | 0.0519 | 0.1421 | 0.1219 | 0.1372 |
| **RH** | 0.1427  (±0.0020) | 0.1327  (±0.0019) | 0.1049  (±0.0016) | 0.0164  (±0.0003) | 0.0000 | 0.0245 | 0.1248 | 0.1070 | 0.1219 |
| **SF** | 0.1227  (±0.0019) | 0.1128  (±0.0018) | 0.0816  (±0.0013) | 0.0519  (±0.0008) | 0.0245  (±0.0004) | 0.0000 | 0.0726 | 0.0802 | 0.0955 |
| **SI** | 0.1495  (±0.0022) | 0.1332  (±0.0021) | 0.1033 (±0.0016) | 0.1421  (±0.0022) | 0.1248  (±0.0019) | 0.0726  (±0.0011) | 0.0000 | 0.0977 | 0.1165 |
| **ER** | 0.1348  (±0.0023) | 0.1173  (±0.0019) | 0.0855  (±0.0014) | 0.1219  (±0.0019) | 0.1070  (±0.0018) | 0.0802  (±0.0014) | 0.0977  (±0.0015) | 0.0000 | 0.0476 |
| **EV** | 0.1528  (±0.0024) | 0.1348  (±0.0021) | 0.1023  (±0.0018) | 0.1372  (±0.0021) | 0.1219  (±0.0020) | 0.0955  (±0.0016) | 0.1165  (±0.0019) | 0.0476  (±0.0011) | 0.0000 |
