# Supplementary material for: Population structure and genomic inbreeding in nine Swiss dairy cattle populations
Source: Genet Sel Evol. 2017 Nov 7;49:83. doi: 10.1186/s12711-017-0358-6 (PMC5674839; doi:10.1186/s12711-017-0358-6)
Supplement: Supplementary file 7 — Additional file 7: Figure S5. Neighbour joining (NJ) tree based on F ST distances. [file 12711_2017_358_MOESM7_ESM.docx]

Figure S5 Neighbour joining (NJ) tree based on $\boldsymbol{F}_{\mathbf{ST}}$ distances
