# Supplementary material for: Population structure and genomic inbreeding in nine Swiss dairy cattle populations
Source: Genet Sel Evol. 2017 Nov 7;49:83. doi: 10.1186/s12711-017-0358-6 (PMC5674839; doi:10.1186/s12711-017-0358-6)
Supplement: Supplementary file 9 — Additional file 9: Figure S7. Distribution of the number of ROH in different length classes and for each population. [file 12711_2017_358_MOESM9_ESM.docx]

Figure S7 Distribution of the number of ROH in different length classes and for each population.
