# Supplementary material for: Population structure and genomic inbreeding in nine Swiss dairy cattle populations
Source: Genet Sel Evol. 2017 Nov 7;49:83. doi: 10.1186/s12711-017-0358-6 (PMC5674839; doi:10.1186/s12711-017-0358-6)
Supplement: Supplementary file 10 — Additional file 10: Table S4. Number of ROH per chromosome and population. [file 12711_2017_358_MOESM10_ESM.docx]

Table S4 Number of ROH per chromosome and population.

| **BTA** | **BS** | **BV** | **OB** | **HO** | **RH** | **SF** | **SI** | **ER** | **EV** | **in total** |
| --- | --- | --- | --- | --- | --- | --- | --- | --- | --- | --- |
| 1 | 350 | 3990 | 130 | 2733 | 1593 | 274 | 259 | 26 | 27 | 9382 |
| 2 | 282 | 2928 | 54 | 2290 | 1144 | 188 | 179 | 17 | 11 | 7093 |
| 3 | 282 | 2663 | 69 | 1361 | 938 | 192 | 113 | 9 | 11 | 5638 |
| 4 | 335 | 3232 | 69 | 1453 | 833 | 181 | 113 | 18 | 14 | 6248 |
| 5 | 104 | 1332 | 29 | 635 | 362 | 78 | 57 | 5 | 9 | 2611 |
| 6 | 436 | 4511 | 64 | 1801 | 1045 | 157 | 155 | 15 | 19 | 8203 |
| 7 | 216 | 2223 | 54 | 1773 | 827 | 134 | 93 | 17 | 14 | 5351 |
| 8 | 310 | 3186 | 109 | 1711 | 942 | 209 | 126 | 21 | 20 | 6634 |
| 9 | 206 | 1941 | 55 | 1097 | 921 | 168 | 133 | 13 | 12 | 4546 |
| 10 | 196 | 2260 | 55 | 2741 | 1220 | 156 | 80 | 14 | 18 | 6740 |
| 11 | 217 | 2489 | 33 | 1093 | 619 | 109 | 56 | 11 | 10 | 4637 |
| 12 | 189 | 2635 | 46 | 860 | 529 | 84 | 54 | 2 | 8 | 4407 |
| 13 | 295 | 3043 | 49 | 1630 | 1028 | 145 | 67 | 13 | 12 | 6282 |
| 14 | 230 | 2725 | 55 | 1743 | 1057 | 198 | 121 | 11 | 3 | 6143 |
| 15 | 158 | 1552 | 43 | 679 | 476 | 81 | 107 | 7 | 9 | 3112 |
| 16 | 127 | 1207 | 23 | 851 | 431 | 67 | 47 | 8 | 7 | 2768 |
| 17 | 216 | 1942 | 53 | 1213 | 700 | 86 | 152 | 11 | 6 | 4379 |
| 18 | 200 | 2128 | 51 | 675 | 1091 | 181 | 75 | 13 | 11 | 4425 |
| 19 | 240 | 2206 | 45 | 1041 | 641 | 90 | 84 | 11 | 19 | 4377 |
| 20 | 120 | 1476 | 49 | 1641 | 957 | 163 | 72 | 11 | 16 | 4505 |
| 21 | 137 | 1636 | 23 | 757 | 510 | 143 | 77 | 6 | 10 | 3299 |
| 22 | 144 | 1898 | 30 | 992 | 806 | 88 | 58 | 7 | 8 | 4031 |
| 23 | 107 | 1063 | 27 | 857 | 428 | 84 | 46 | 1 | 7 | 2620 |
| 24 | 144 | 1514 | 27 | 1224 | 558 | 76 | 61 | 14 | 4 | 3622 |
| 25 | 163 | 1640 | 32 | 954 | 614 | 80 | 86 | 7 | 8 | 3584 |
| 26 | 105 | 1175 | 56 | 856 | 419 | 83 | 63 | 1 | 8 | 2766 |
| 27 | 128 | 1269 | 16 | 582 | 391 | 108 | 72 | 3 | 8 | 2577 |
| 28 | 118 | 1482 | 24 | 641 | 373 | 79 | 53 | 12 | 8 | 2790 |
| 29 | 137 | 1437 | 12 | 614 | 526 | 90 | 44 | 1 | 9 | 2870 |
| in total | 5892 | 62,783 | 1382 | 36,498 | 21,979 | 3772 | 2703 | 305 | 326 | 135,640 |
