# Supplementary material for: Population structure and genomic inbreeding in nine Swiss dairy cattle populations
Source: Genet Sel Evol. 2017 Nov 7;49:83. doi: 10.1186/s12711-017-0358-6 (PMC5674839; doi:10.1186/s12711-017-0358-6)
Supplement: Supplementary file 11 — Additional file 11: Figure S8. Regression of F PED on F HOM for all 9214 individuals. Multiple R-squared: 0.45. [file 12711_2017_358_MOESM11_ESM.docx]

Figure S8 Regression of F_PED on F_HOM for all 9214 individuals. Multiple and adjusted R–squared: 0.45
