# Supplementary material for: Population structure and genomic inbreeding in nine Swiss dairy cattle populations
Source: Genet Sel Evol. 2017 Nov 7;49:83. doi: 10.1186/s12711-017-0358-6 (PMC5674839; doi:10.1186/s12711-017-0358-6)
Supplement: Supplementary file 12 — Additional file 12: Table S5. Correlations between F PED and F ROH and between F PED and F HOM including all animals. Table S6. Correlations between F PED and F ROH and between F PED and F HOM including only animals with PCI > 0.95. [file 12711_2017_358_MOESM12_ESM.docx]

Table S5 Correlations including all animals.

|  | **Total (all animals)** | **BS** | **BV** | **OB** | **HO** | **RH** | **SF** | **SI** | **ER** | **EV** |
| --- | --- | --- | --- | --- | --- | --- | --- | --- | --- | --- |
| r(F_PED_,F_ROH_) | 0.699 | 0.543 | 0.637 | 0.592 | 0.609 | 0.659 | 0.755 | 0.741 | 0.376 | 0.402 |
| r(F_PED_, F_HOM_) | 0.673 | 0.600 | 0.669 | 0.588 | 0.648 | 0.644 | 0.752 | 0.754 | 0.293 | 0.497 |

Table S6 Correlations including only animals with PCI >0.95.

|  | **Animals with PCI >0.95** | **BS animals with PCI >0.95** | **BV animals with PCI >0.95** | **OB animals with PCI >0.95** | **HO animals with PCI >0.95** | **RH animals with PCI >0.95** | **SF animals with PCI >0.95** | **SI animals with PCI >0.95** | **ER animals with PCI >0.95** | **EV animals with PCI >0.95** |
| --- | --- | --- | --- | --- | --- | --- | --- | --- | --- | --- |
| r(F_PED_,F_ROH_) | 0.701 | 0.522 | 0.613 | 0.569 | 0.631 | 0.657 | 0.747 | 0.734 | 0.191 | – |
| r(F_PED_, F_HOM_) | 0.677 | 0.589 | 0.644 | 0.562 | 0.672 | 0.657 | 0.747 | 0.745 | 0.113 | – |
