# Supplementary material for: Population structure and genomic inbreeding in nine Swiss dairy cattle populations
Source: Genet Sel Evol. 2017 Nov 7;49:83. doi: 10.1186/s12711-017-0358-6 (PMC5674839; doi:10.1186/s12711-017-0358-6)
Supplement: Supplementary file 14 — Additional file 14: Figure S10.. Genomic distribution of the d i statistic for all 1-Mb windows across all autosomes and the eight groups of populations. The dashed red line denotes the 99th percentile for each population group (BS: Brown Swiss, BV: Braunvieh, OB: Original Braunvieh; HO: Holstein, RH: Red Holstein, SF: Swiss Fleckvieh, SI: Simmental, ER/EV: Eringer and Evolèner). [file 12711_2017_358_MOESM14_ESM.docx]

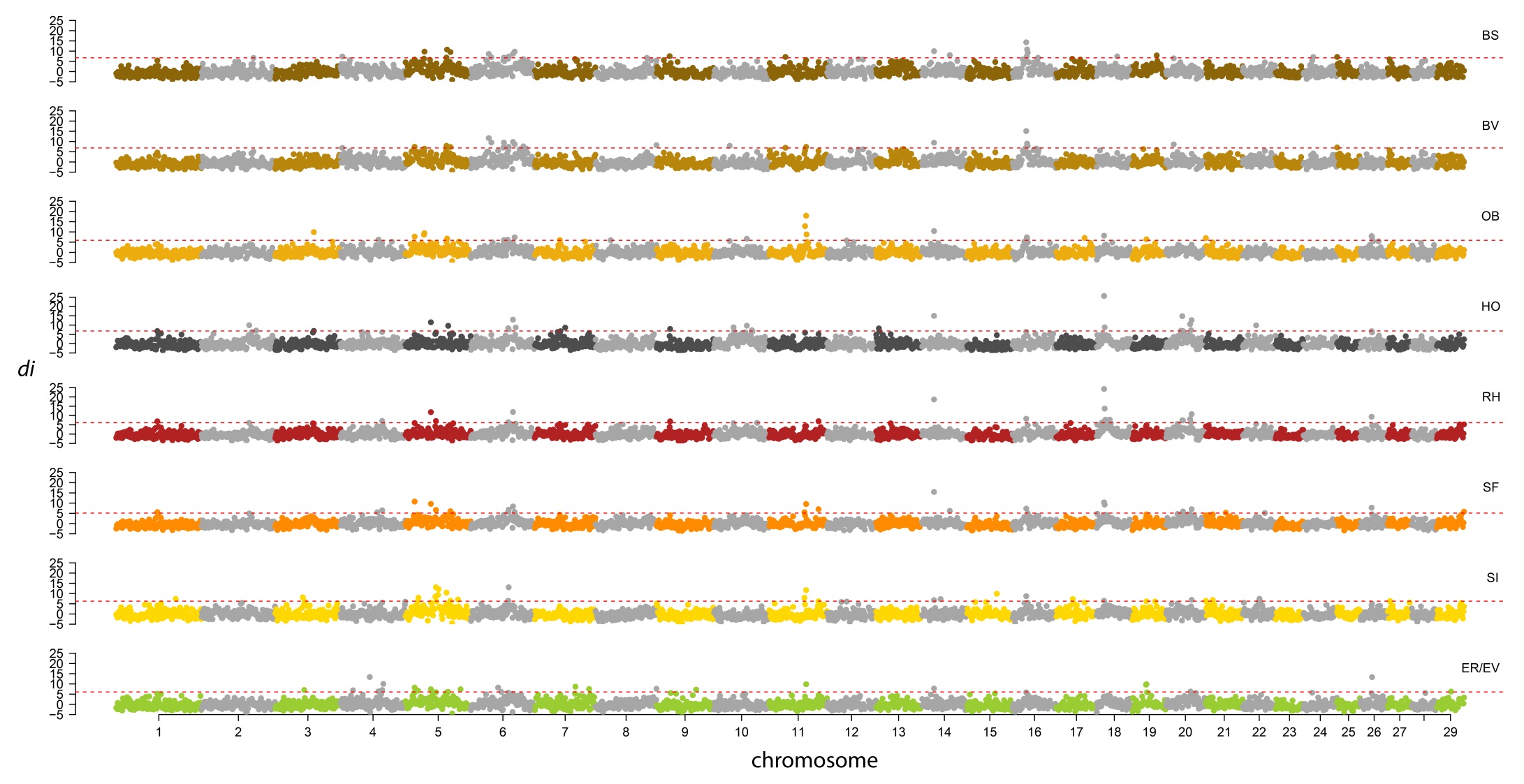


Figure S10 Genomic distribution of the $\boldsymbol{d}_{\boldsymbol{i}}$ statistic for all 1-Mb windows across all autosomes and the eight groups of populations. The dashed red line denotes the 99th percentile for each population group (BS: Brown Swiss, BV: Braunvieh, OB: Original Braunvieh; HO: Holstein, RH: Red Holstein, SF: Swiss Fleckvieh, SI: Simmental, ER/EV: Eringer and Evolèner).
